# Neonatal Anesthesia and Oxidative Stress

**DOI:** 10.3390/antiox11040787

**Published:** 2022-04-16

**Authors:** David A. Gascoigne, Mohammed M. Minhaj, Daniil P. Aksenov

**Affiliations:** 1 Department of Radiology, NorthShore University HealthSystem, Evanston, IL 60201, USA; dgascoigne@northshore.org; 2 Department of Anesthesiology, NorthShore University HealthSystem, Evanston, IL 60201, USA; mminhaj@northshore.org

**Keywords:** reactive oxygen species, antioxidants, GABA, glutamate, neurovascular unit, development, propofol, sevoflurane, isoflurane, ketamine

## Abstract

Neonatal anesthesia, while often essential for surgeries or imaging procedures, is accompanied by significant risks to redox balance in the brain due to the relatively weak antioxidant system in children. Oxidative stress is characterized by concentrations of reactive oxygen species (ROS) that are elevated beyond what can be accommodated by the antioxidant defense system. In neonatal anesthesia, this has been proposed to be a contributing factor to some of the negative consequences (e.g., learning deficits and behavioral abnormalities) that are associated with early anesthetic exposure. In order to assess the relationship between neonatal anesthesia and oxidative stress, we first review the mechanisms of action of common anesthetic agents, the key pathways that produce the majority of ROS, and the main antioxidants. We then explore the possible immediate, short-term, and long-term pathways of neonatal-anesthesia-induced oxidative stress. We review a large body of literature describing oxidative stress to be evident during and immediately following neonatal anesthesia. Moreover, our review suggests that the short-term pathway has a temporally limited effect on oxidative stress, while the long-term pathway can manifest years later due to the altered development of neurons and neurovascular interactions.

## 1. Introduction

General anesthesia is the standard of care for a vast array of procedures and surgeries in neonates. While approximately 650,000 children born each year in the USA will be exposed to some form of general anesthesia before their third birthday [1], the fundamental mechanisms and alterations to neural activity that form the foundation of general anesthesia are not fully understood [2]. What is known is that anesthetic agents reduce and disrupt neuronal activity across the central nervous system (CNS) to levels that are insufficient to support conscious awareness.

There are a variety of anesthetic agents, each with a unique pharmacokinetic and pharmacodynamic profile, that are currently in use for children, further complicating our understanding of how general anesthesia affects the developing brain. The literature demonstrates a range of harmful consequences that have been reported. Some of these are short-lived and do not tend to incur any lasting effects, such as nausea, vomiting, emergence delirium, and minor airway complications [3,4,5]. Importantly, however, long-term consequences have also been reported. Specifically, learning and behavior deficits have been observed in later childhood following neonatal anesthesia exposure. These effects were first reported by a series of retrospective observational studies. An example of which was a study conducted by Wilder et al. [6], which analyzed learning disabilities in children exposed to anesthesia before the age of 4. The investigators found a significant direct relationship between the duration of anesthesia and the likelihood of a diagnosed learning disability. Another example can be seen in an article by Chemaly et al. [7], which identified a similar relationship between anesthesia exposure in young children and abnormal behavior in later childhood. Subsequent reviews and reports have provided converging evidence for the observed delayed effects of neonatal anesthesia [8,9,10], prompting mechanistic interrogation using animal models.

Animal models have primarily been used for this purpose as they are accompanied by an array of benefits that are not otherwise available, such as controlled experimental manipulation, histological analysis of affected tissues, and standardization of methods. Repeatedly, animal models of neonatal anesthesia have reported neuroapoptosis, neurotoxicity, and learning/behavioral changes following neonatal anesthesia exposure [11]. Moreover, the severity of damage caused by anesthesia has shown to be dose and duration dependent, such that higher dosages and durations are accompanied by more profound consequences [12].

In light of the results of these retrospective and animal studies, several prospective studies have subsequently been performed in children. The Pediatric Anesthesia Neurodevelopment Assessment (PANDA), the Mayo Anesthesia Safety in Kids (MASK) study, and the General Anesthesia Spinal (GAS) study are well known examples. These investigations were predominantly limited to short durations of anesthesia in children and indicated longer durations to be necessary for later development effects to be visible—a notion that has been supported by additional research in children [13,14]. Thus, both human and animal models of prolonged neonatal anesthesia provide converging evidence of the associated long-term risks of neonatal anesthesia.

It has been postulated that the observed learning and behavior deficits that result from neonatal anesthesia manifest due to a range of neuronal connectivity and neurovascular alterations, such as neuroapoptosis across brain structures, changes in the excitatory/inhibitory balance, and debilitated neurovascular coupling [10]. However, how anesthesia enacts these changes is poorly understood. One of the leading hypotheses is that initial oxidative stress can engender neuroapoptosis [15], which can induce a cascade of subsequent debilitating neurological effects. When these conditions occur, the production of reactive oxygen species (ROS) escalates. Typically, ROS are a normal byproduct of brain cell metabolism; however, their accumulation in oxidative stress can overwhelm the natural protective antioxidant capacity of the brain, leading to cell debilitation and death.

In this review, we focus on the different pathways by which anesthesia may produce oxidative stress both intraoperatively and postoperatively. Before we address this, we provide a brief overview of common anesthetics that are currently in clinical use and/or have been used in translational research to better understand the effects of neonatal anesthesia.

## 2. Actions of Common Anesthetics

Anesthetic agents can be organized into two main groups—volatile and nonvolatile anesthetics. Volatile anesthetics are liquid at room temperature and are inhaled with the assistance of a vaporizer [16]; nonvolatile anesthetics are typically administered via intravenous (IV) or intramuscular (IM) injections and titrated or repeated to achieve the desired level of anesthesia [17,18,19]. We will focus on representative examples of the two categories that have been used for neonatal anesthesia research. Isoflurane and sevoflurane are discussed for volatile anesthetics, and propofol and ketamine are the focus of nonvolatile anesthetics.

### 2.1. Volatile Anesthetics

Sevoflurane is the most common volatile anesthetic used in both adults and children [20]. Isoflurane is used mostly in research and occasionally in clinical practice. These agents are administered via inhalation, almost always with supplemental oxygen, at varying volume concentrations and have notable overlaps in their mechanisms of action. Specifically, both of these anesthetics are known to reversibly bind to postsynaptic gamma-aminobutyric acid (GABA) receptors, enhancing the primary inhibitory neurotransmitter in the CNS, GABA, and induce hyperpolarization by allowing an influx of chloride ions into the cell [21,22]. Volatile anesthetics achieve this by increasing GABA receptor sensitivity to GABA and by prolonging its effect [22]. Moreover, isoflurane and sevoflurane have also shown to activate presynaptic potassium channels [23], providing another mechanism by which they elicit hyperpolarization and, in turn, neuronal inhibition.

These inhibitory effects, which produce the desired level of sedation, is characterized by a rapid onset and termination of action due to its low levels of hepatic metabolism [24]. Instead of requiring hepatic and renal metabolism of the anesthetic to clear the agent from the body, it can predominantly be exhaled. The similarities in the mechanisms of action of these small fluorinated hydrocarbons can be attributed to their similar molecular structures [16]; however, the subtle differences in their structure and composition can account for their biochemical differences.

The largest dissociation in the effect of isoflurane and sevoflurane can be seen in their inverse effect on presynaptic glutamate release. In vitro studies have found polar opposite effects of isoflurane and sevoflurane on glutamate suppression. Initially, Larsen et al. [25] identified an inverse relationship between calcium-dependent glutamate suppression and isoflurane concentration, and a subsequent investigation, by Vinje et al. [26], found a direct relationship between glutamate-suppression and sevoflurane concentrations. Strikingly, as the concentration of these anesthetics increase, their respective suppressive impacts on glutamate release shift in opposite directions. These differences can explain in vivo findings that demonstrate lower suppression of single-unit activity by 1 minimum alveolar concentration (MAC) isoflurane when directly compared with 1 MAC sevoflurane [27].

### 2.2. Nonvolatile Anesthetics

Nonvolatile anesthetics can vary much more drastically in terms of both their method of administration and their mechanisms of action. Propofol is an anesthetic agent that first became commercially available in the United States in 1989 [28] and has since become one of the most commonly utilized IV anesthetics in its emulsified formulation [18,29]. A significant amount of evidence suggests that propofol primarily acts by interacting with ligand-gated GABA receptors [30]. By enacting such increases in inhibitory activity in areas of the CNS associated with consciousness (e.g., thalamus [31,32], cerebral cortex [33,34,35], brain stem [36,37]), propofol produces its characteristic hypnotic effects.

In terms of neuronal circuits, propofol seems to induce unconsciousness in a unique manner. This was elucidated by a study that combined simultaneous single-unit, local field potential, and intracranial electrocorticogram recordings while human subjects transitioned into an unconscious state from propofol [38]. The study identified that unconsciousness almost immediately followed the onset of slow local field potential oscillations and decreases in neuronal activity. The authors suggested that propofol’s neurophysiological mechanism of eliciting anesthesia involves spatially and temporally fragmenting cortical activity and, therefore, disrupting the functional connectivity in the cerebral cortex.

Propofol metabolism is vastly different from that of volatile anesthetics. While isoflurane and sevoflurane can be exhaled with minimal hepatic intervention, propofol heavily relies on the function of the liver and the kidneys [39]. Propofol’s use in children is supported by clinical trials from the 1990s, which demonstrated its safety for the induction and maintenance of anesthesia [40,41]. The primary benefits of propofol are its relatively quick action for an IV anesthetic [42] and its dose dependency, such that it may be used as a sedative for nonsurgical interventions in lower doses. However, adverse effects of sole propofol administration have been noted, including respiratory depression and hypotension [43,44,45]. To combat these effects, ketamine administration in combination with propofol for sedation can be performed [19], although its efficacy in reducing common short-term adverse effects has delivered mixed results [46,47].

Ketamine, an N-methyl-D-aspartate (NMDA) receptor antagonist, is not typically used as a monotherapeutic agent in adults or children, although it has been shown to be useful in children for achieving a range of applications, such as anesthesia, sedation, analgesia, and antidepressant treatment. This is due to its relatively small effect on blood pressure, respiration rate, and airway reflexes [48]. Moreover, when ketamine is administered intraoperatively following propofol-induced anesthesia, it has shown to reduce the consumption of opioid analgesics; lower pain intensity, both immediately and 6 weeks after major surgery; and increase patient satisfaction [49,50].

Typically, NMDA receptors facilitate the effusion of calcium ions (Ca^2+^) out of the cell, and thus the depolarization of neurons, in the presence of glutamate. In addition to inhibiting presynaptic glutamate release [51], ketamine achieves the reduction of excitatory activity by allosterically binding to the NMDA receptors to lower the frequency and duration at which these receptors are active [49]. Secondarily to its NMDA receptor antagonism, ketamine is also known to interact with opioid, cholinergic, nicotinic, muscarinic, and monoaminergic receptors. In children, the pharmacokinetics of ketamine is characterized by increased intramuscular absorption and faster hepatic metabolism and clearance of the drug from the body [52,53].

## 3. Oxidative Stress from Neonatal Anesthesia

### 3.1. Oxidative Stress and the Antioxidative Defense System in the Brain

The health of neuronal cells directly relies on the concentration of ROS, which can either support neuronal health in physiological concentrations or be incredibly harmful in severely elevated concentrations. The most common ROS are superoxide (O_2_^−^), hydrogen peroxide (H_2_O_2_), hydroxyl radicals (HO•), nitric oxide (NO), lipid peroxide, singlet oxygen (^1^O_2_), and peroxynitrite (ONOO^−^). Note that certain ROS can participate in cell signaling, growth, repair, gene expression, and motor activity [54] and are produced via multiple essential processes, including the mitochondrial electron transport chain, the endoplasmic reticulum, and various enzymes (e.g., NADPH oxidase, xanthine oxidase, nitric oxide synthase, lipoxygenase) [55]. Normal cell metabolism relies on oxidative phosphorylation in the mitochondria to produce adenosine triphosphate (ATP). A natural byproduct of this process is the generation 85–90% of the body’s ROS [56].

Depending on the cell type, the production of ROS accounts for up to 11% of oxygen consumption in the presence of an altered antioxidant system [57] (the natural protective system against oxidative stress). In comparison, under normal conditions, the production of ROS is approximately 2% of metabolized oxygen [58]. A shift in the balance between ROS and their antioxidant counterparts in favor of ROS over antioxidants is the definition of oxidative stress and has been associated with a range of pathologies [59]. 

Below, we review the most common ROS, in greater detail, and those that are specific to the consequences of neonatal anesthesia. This is followed by the prominent components of the antioxidative defense system in the brain.

#### 3.1.1. Superoxide

Superoxide is one of the most abundant ROS. It is generated by both spontaneous and enzymatic activity [60,61], and its most likely source is the mitochondrion. This method of production of superoxide occurs in the inner membrane of mitochondria, where electrons that leak from the electron transfer system lead to the formation of superoxide. These electrons are captured by molecular oxygen (O_2_) and become O_2_^−^ [62]. The superoxide anion theoretically can participate in the iron-catalyzed Haber–Weiss reaction, which generates highly reactive HO• [63]. In this reaction, the role of superoxide is to reduce Fe^3+^ to Fe^2+^. However, O_2_^−^ is often outcompeted by other reductants (e.g., glutathione, ascorbate, and NADPH) due to poor chemical reactivity [64]. It seems that superoxide can act on specific intracellular targets (for example, by inactivation of enzymes), and its effect includes indirect damage to DNA [64]. Superoxide is able to produce HO• by oxidizing [4Fe-4S]-clusters of dehydratases that release Fe^2+^, and it also reduces H_2_O_2_ [65]. Moreover, superoxide can oxidize various targets, such as sulfite, thiols, leukoflavins, and several enzymes [66]. It is important to note that superoxide reacts with nitric oxide (NO•) (O_2_^−^ + NO = ONOO^−^) [67] and forms peroxynitrite with a diffusion-controlled rate, because NO• has an unpaired electron [62].

An animal model of neonatal anesthesia has shown a greater than 100% increase in superoxide concentrations in the brain and nearly a 40% increase in superoxide catalyzing NADPH oxidase [68]. Moreover, these effects were associated with long-term memory impairment—consistent with other findings of neonatal anesthesia.

#### 3.1.2. Hydrogen Peroxide

Hydrogen peroxide and superoxide are the two most common ROS. Hydrogen peroxide is the most stable endogenous ROS and is a byproduct of multiple metabolic and signaling pathways [69]. Two major sources of intracellular H_2_O_2_ exist: (1) the leaking of electrons from the mitochondrial electron transport system and (2) NADPH oxidases that transform oxygen into superoxide (O_2_^−^) for subsequent H_2_O_2_ formation, which is catalyzed by superoxide dismutase (SOD) [70]. H_2_O_2_ itself can react with certain macromolecules in the cytoplasm, or it can break down to form the most reactive radical of ROS, HO• [69]. This can happen via the Fenton reaction, where H_2_O_2_ reacts with iron cations (Fe^2+^ and Fe^3+^), yielding a hydroxide ion and HO•. The typical pathways for removing intracellular H_2_O_2_ include regulatory enzymes (catalase, peroxiredoxins, glutathione peroxidases, etc.) as well as the antioxidant molecules, such as vitamin C and α-ketoacids [70]. Note that H_2_O_2_ and NO do not react to form peroxynitrite [67]. Under anesthesia, since H_2_O_2_ is a byproduct of superoxide reacting with SOD, the concentration of H_2_O_2_ is an indirect indicator of the effectiveness of this process and the anesthesia-induced concentration of superoxide [71].

#### 3.1.3. Hydroxyl Radical

The hydroxyl radical (HO•) can be generated by the Fenton reaction between H_2_O_2_ and Fe^2+^; by homolysis of H_2_O_2_, the fission of H_2_O upon exposure to ionizing radiation; and perhaps by homolysis of the excited water molecule (for a review of the likelihood and conditions of these reactions to occur in vivo, see [72]). Hydroxyl radicals can cause oxidative damage to almost every organic biomolecule, which leads to the impairment of normal cellular functioning [72]. Anesthetics have shown to have had differing effects on the rate of hydroxyl radical production. Propofol has shown to help lower the degree of oxidative stress from hydroxyl radical formation [73], which has been proposed to be a result of its similar molecular structure to ROS scavengers, such as vitamin E [74]. However, inhalation anesthetics, such as sevoflurane, have not shown to have such an effect [75].

#### 3.1.4. Nitric Oxide

NO is an unstable and highly lipophilic gas, which is synthesized by nitric oxide synthase (NOS) from molecular oxygen and L-arginine in the presence of several cofactors: NADPH, FMN, FAD, heme, and BH_4_ [76]. There are three isoforms of NOS: neuronal (nNOS), endothelial (eNOS), and inducible (iNOS). Neuronal and endothelial NOS are expressed due to normal physiological mechanisms, and inducible NOS is expressed in glia in response to stress, inflammation, or neurodegeneration [77]. All neurons can express nNOS; however, the effectiveness of this process is age and region dependent. There is an established pathway for nNOS generation in neurons: glutamate binds to NMDA receptors, which triggers the influx of Ca^2+^ through the channel that binds calmodulin and activates nNOS, which starts the synthesis of NO [76]. NO is known to have vasodilatory properties and cause the activation of soluble guanylate cyclase (sGC), which triggers the production of cyclic guanosine monophosphate (cGMP) [77,78]. Activation of protein kinase G (PKG) by cGMP induces a decrease in Ca^2+^, which leads to the dephosphorylation of the myosin light chain and smooth muscle cell relaxation [76]. Although the physiological production of NO• is transient, pathophysiological longer-term production of NO• can lead to increasingly cumulative and irreversible protein 3-nitrotyrosination [77]. During anesthesia, particularly when volatile anesthetics are administered, cerebral vasodilation is known to occur [79], and this has often been attributed to the involvement of elevated levels of neurally derived nitric oxide in response to anesthetic agents [80]. A higher concentration of NO reacts with O_2_•^−^ to generate a large amount of peroxynitrite (ONOO^−^), which is a highly cytotoxic ROS because it aggressively oxidizes proteins, lipids, and DNA [71].

#### 3.1.5. Peroxynitrite

Superoxide can react with NO• and form ONOO^−^, a potent biological oxidant that participates in ROS-induced tissue injury. Peroxynitrite directly reacts with target biomolecules (membrane lipids, thiols, proteins, and DNA) via one- or two-electron oxidations [81]. The reaction of ONOO^−^ with membrane lipids (even without iron) induces a phospholipid membrane peroxidation product. When lipid hydroperoxides break down, aldehydes are generated from the peroxidation of ω-6 unsaturated fatty acids (arachidonic acid and linoleic acid), producing 4-hydroxynonenal (HNE), which is a very toxic and potent nine-carbon α, β-unsaturated aldehyde. Peroxynitrous acid (a protonated form of ONOO^−^:ONOOH) produces highly reactive HO• and nitrogen dioxide radicals (NO_2_•), which cause oxidation and nitration [62]. Another important mechanism of cellular injury is a ONOO^−^-dependent increase in DNA strand breakage, which triggers the activation of a DNA repair enzyme (PARP). DNA damage leads to the overactivation of PARP, which induces the depletion of oxidized nicotinamide adenine dinucleotide (NAD) and ATP, causing necrotic cell death [81]. Anesthesia does not seem to directly influence the rate of peroxynitrite production, but instead increases the abundance of the reactants in its production. However, propofol has shown to attenuate some of the cellular damage associated with excess peroxynitrite [82].

#### 3.1.6. Antioxidant Defense

The antioxidative system consists of enzymatic and nonenzymatic antioxidants. In short, it includes reductants and their cofactors that react with ROS and free radicals, thereby preventing their accumulation and neutralizing their toxic effects. Moreover, the functionality of the antioxidant system extends to the repair and removal of affected biomolecules to maintain normal cell metabolism [83,84,85].

The primary enzymatic antioxidants in the brain are superoxide dismutase, catalase, glutathione peroxidase, and thioredoxins. Superoxide dismutase, for example, helps transform the superoxide radical into a less reactive H_2_O_2_ molecule, which, in turn, can protect dehydratases, a group of enzymes essential in metabolism and cell function by forming bonds with the elimination of water molecules, from oxidation [86]. A specific example is manganese superoxide dismutase (MnSOD), which inhibits the formation of ONOO^−^ by decreasing superoxide levels [62]. In humans, there are three isoforms of superoxide dismutase, and they often require activation by a catalytic metal. It has been shown that general anesthesia decreases the levels of SOD [87] and the possible mechanism maybe related to the anesthesia-induced overexpression of sirtuin 3 (SIRT3), which is a nicotinamide adenine dinucleotide (NAD)-dependent deacetylase and plays a key role in regulating mitochondrial dysfunction [88]. Glutathione peroxidase, on the other hand, facilitates the reduction of hydroperoxides (including H_2_O_2_), yet it is less effective than catalase under more severe oxidative stress. It is interesting that in a nonmammal model, glutathione peroxidase was affected much less by anesthesia than SOD, and postanesthesia increase in glutathione peroxidase in the recovery period could reflect just a normal response to the increased ROS production [89]. Catalase, a heme-containing protein, also reacts with H_2_O_2_ molecules to yield water and molecular oxygen via disproportionation, but does not become saturated by H_2_O_2_, even in cases of severe oxidative stress, due to the extreme rate of hydrogen peroxide dismutation [90,91]. In the same nonmammal model brain, catalase was not affected by anesthesia [89]. These three enzymatic antioxidants are not an exhaustive list of intracellularly generated antioxidants. Indeed, heme oxygenase-1, thioredoxins, peroxiredoxins, and glutaredoxins can play important roles in the body, yet superoxide dismutase, glutathione peroxidase, and catalase do compose the major enzymatic antioxidant defense in the brain.

The functions of nonenzymatic antioxidants in the brain can be far more diverse. For example, they can quench singlet oxygen, inhibit oxidative enzymes, decompose peroxide species, and absorb ultraviolet radiation [92]. Selenium and zinc are common elemental antioxidant enzyme cofactors. Selenium’s antioxidative properties are typically attributed to its role in regulating Ca^2+^ signaling, thus contributing to enzymatic antioxidants as a cofactor [93]. Zinc plays a similar role as it primarily acts to increase the activity of the major enzymatic antioxidants, but it can also debilitate oxidant-producing enzymes [83,94]. Zinc deficiency has shown to be strongly associated with oxidative stress [95]. Perhaps the levels of nonenzymatic antioxidants are more resistant to anesthesia than SOD [89].

In conjunction with these pro-antioxidants, water- and lipid-soluble vitamins exist. Ascorbic acid (vitamin C), a water-soluble antioxidant, is able to cross the blood–brain barrier (BBB) and diffuse into neurons with the facilitation of sodium ascorbate transporter proteins [96]. Ascorbic acid is known to be involved in antioxidation as either an agent or a cofactor [97]. When ascorbic acid protects hydrophobic cell entities (e.g., membranes, lysosomes), it often operates in conjunction with a lipid soluble component of the antioxidant defense system, vitamin E [98,99]. α-Tocopherol, the most biologically active form of vitamin E, is a chain-breaking antioxidant, which predominantly works to protect the integrity of cell membranes against polyunsaturated fatty acid-generated ROS [100,101]. Another important lipid-soluble nonenzymatic component of the antioxidant defense system is vitamin A. Retinol and β-carotene are common forms and are a commonly ingested micronutrient from both animal and plant products. Specifically, β-carotene is often more effective than α-tocopherol when protecting against hydrophobic radicals [83]. Evidence suggests the most effective protection of membranes against ROS occurs when α-tocopherol and ascorbic acid work in tandem [102].

These components of the antioxidant defense system are relatively omnipresent throughout the body. Unfortunately, the brain has a weak endogenous antioxidant defense system in comparison with other organs [83], rendering it uniquely susceptible to toxicity. The reason for this is a lower concentration of antioxidants in comparison with high metabolic demand, which permits levels of ROS to rapidly accumulate far beyond homeostatic levels. Evidently, the fragility of redox balance in the brain leads to its susceptibility to detrimental insults, such as oxidative stress.

In order to understand oxidative stress from neonatal anesthesia, the discrepancies between the adult and neonatal antioxidant defense system should first be noted. An animal model has shown that the immature brain has limited glutathione peroxidase activity and is very susceptible to oxidative stress [103]. Moreover, another study indicated that in the neonatal brain, overexpression of SOD1 is deleterious since there is no compensation for this from increased activities of downstream enzymes (glutathione peroxidase and catalase) [104]. These findings corroborate the notion that superoxide dismutase and glutathione peroxidase appear to provide the most substantial contribution to the neonatal antioxidant defense system, which has been supported in numerous other studies that have primarily been conducted by simulating the activity of these enzymes with mimicking agents [105,106,107,108]. Studies in humans have shown that full-term infants are susceptible to oxidative stress, which only resolves with age [109] and reflects the transient imbalance between ROS and the antioxidative system. Thus, with less respondent antioxidants, the neonatal brain has a heightened susceptibility to the effects of oxidative stress.

### 3.2. Oxidative Stress during Anesthesia

**Figure 1:**
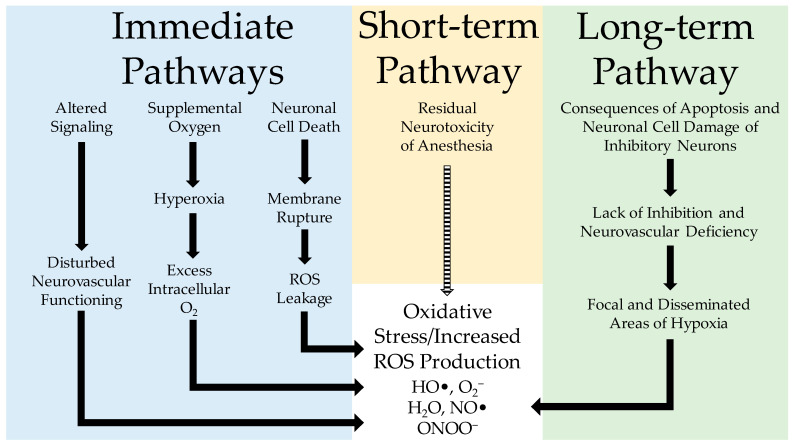
Schematic illustration of neonatal anesthesia-induced oxidative stress in the brain. During the administration of neonatal anesthesia, there are three immediate pathways (blue) for how oxidative stress (red) can manifest. These immediate pathways have three distinct sources, altered signaling, administration of supplemental oxygen, and neuronal cell death; each of these can increase the concentration of ROS and induce oxidative stress. The short-term pathway (yellow) appears to be less impactful since it is short-lived; this is shown in the figure with a dashed arrow. The long-term pathway (green) shows the delayed effects of neonatal anesthesia and how the initial damage to neurons can negatively affect neurovascular interactions, leading to oxidative stress.

During the course of anesthesia, apoptosis, altered signaling, and potentially hyperoxic conditions are known to occur. Each of these primary factors is known to elevate the production of ROS, thereby producing oxidative stress (Figure 1).

Reduced respiratory rate and volume are some of the leading concerns of anesthesiologists when caring for a patient under anesthesia, as it is a prolific and expected consequence of most of the aforementioned agents. In an effort to combat a potential state of hypoxia that would result from lowered blood oxygenation, supplemental oxygen is almost universally administered in concentrations ranging from 30% to 100% O_2_ [110]. While supplemental oxygen has very high efficacy in preventing hypoxia, it can engender hyperoxic conditions in the brain, which are known to increase the generation of ROS (most notably hydrogen peroxide, hydroxyl radicals, and superoxide). In the awake state, the level of inspired oxygen can be self-regulated by a reduction in respiration volume and rate to maintain homeostatic levels of percent oxygen in the blood (PO_2_) [111], and tissue oxygen levels throughout the whole brain can be controlled by cerebrovascular autoregulation in response to fluctuations in arterial pressure (for an in-depth review, see [112]). However, effective self-regulation is not possible in the anesthetized state [8]. Thus, providing supplemental oxygen presents the risk of hyperoxia. Increasing the fraction of inspired O_2_ from 0.21 (normal) to 1.0 (100% O_2_) leads to a fivefold increase in dissolved O_2_ [113]. This increased oxygenation of blood has an exacerbated impact on brain tissue oxygen levels, as one study has shown. Through the use of direct recording of brain tissue PO_2_ in the cortex of neonatal rabbits, the authors observed up to a 300% increase in PO_2_ when the rabbits were administered 80% O_2_ and 1 MAC isoflurane, compared with awake levels [8]. Similar changes were also observed under 1 MAC sevoflurane. The increase in brain tissue PO_2_ illustrates a direct pathway of hyperoxia-associated oxidative stress in the brain for the range of anesthetic agents that are accompanied by concurrent supplemental oxygen.

Anesthesia has a direct and profound impact on neuronal signaling and the electrophysiological behavior of neurons. That is, propofol, isoflurane, and sevoflurane, in addition to the known reduction of global activity, can evoke burst suppression in high concentrations [114,115], which is likely a contributing factor to the resulting anesthetized state. Burst suppression is often noted on electroencephalography (EEG) recordings by its characteristic quasiperiodic and brief phases of extensive neuronal firing (burst), followed by considerably longer latency periods of minimal activity (suppression). Bursts of neuronal firing result in increased metabolic demand, which can rapidly deplete the locally available oxygen because general anesthesia disturbs normal neurovascular coupling [116]. Thus, it has been proposed that these events may be one of the sources of anesthesia-induced oxidative stress [117]. Interestingly, the burst suppression that is observed under anesthesia shares almost identical electrophysiological characteristics with multiple neuropathologies that are comorbid with neuronal cell death, such as, hypoxia, hypothermia, coma, and infantile early epileptic encephalopathy [117].

The administration of anesthesia itself is consistently accompanied by neuroapoptosis, particularly in the neonatal population. Both in vitro and in vivo analyses of the effects of volatile anesthetics as well as propofol and ketamine have elucidated their tendency to induce neurotoxicity in brain tissue. Animal models of anesthesia-related neuroapoptosis and neurotoxicity are the most prolific in demonstrating this phenomenon [118,119,120,121,122,123,124,125,126]. There is a wide spectrum of contributing factors that likely participate in the activation of neuroapoptosis during anesthesia. For example, one hypothesis of the etiology of the widespread apoptosis is focal areas of oxidative stress [127] in anesthetics with glutamatergic properties. This hypothesis-driven model proposes that after excessive activation of NMDA receptors, the buffering capacity of mitochondria no longer responds to a Ca^2+^ overload, which results in an increase of ROS. Additionally, the dissociation of some transcription proteins (e.g., NF-kappaB) occurs. These transcription factors bind to DNA in the nucleus, which results in an imbalance between antiapoptotic heterodimers and proapoptotic homodimers. The latter produces mitochondrial pores that allow the diffusion of cytochrome c into the cytoplasm and activation of caspases, leading to apoptosis.

Another theory involves the effects of inhalation anesthetics on the intracellular calcium homeostasis via the release of calcium from the endoplasmic reticulum, thereby leading to neuronal cell damage [128]. One study identified that isoflurane, sevoflurane, and desflurane all induce significant decreases in endoplasmic reticulum calcium concentrations and neuronal cell damage, except in cells that have total 1,4,5-triphosphate (IP_3_) receptor knockout. These results support increased calcium signaling through IP_3_ receptors in the presence of volatile anesthetics—a process that is known to increase apoptotic sensitivity [129].

In addition, GABA-specific mechanisms have also been researched. Studies have shown that the neuroapoptosis that results from neonatal anesthesia can have a disproportionately high impact on GABAergic interneurons [130,131], which is accompanied by the downregulation of central GABA-synthesizing enzymes, GAD65 and GAD67 [132]. This illustrates a possible toxic effect of anesthetics on the inhibitory system, which would, in turn, have further downstream consequences, affecting cells that GABAergic interneurons help regulate. It is yet to be determined whether the changes in GABA-producing enzymes were observed due to a reduction in GABAergic neurons or by anesthesia-induced downregulation of the enzyme. However, Istaphanous et al. reported a 50% reduction in GAD67 and over 90% reduction in GAD65 concentrations following neonatal anesthesia [132]. Considering this imbalance in reduction, in conjunction with the fact that GAD65 is typically located in nerve terminals, suggests that there can be an additional effect of cellular damage to the surviving cells.

An interesting mechanism of anesthesia-induced apoptosis involves both glutamatergic and GABAergic pathways. Yang et al. suggested that anesthetics affecting GABA and glutamate receptors inhibit brain-derived neurotrophic factor (BDNF) [133]. This biomolecule, BDNF, plays an integral role in the survival and differentiation of neurons throughout development [134], and its dysfunction has shown to be comorbid with a range of neuropathologies that are characterized by learning and memory deficits [135]. Given BDNF’s essential role in the maintenance and functioning of cells, it is clear how negative BDNF-anesthesia interactions could lead to apoptosis in a range of brain structures.

Oxidative stress, as a result of elevated ROS, is known to initiate apoptotic pathways. While apoptosis is deemed to be relatively insular, it is not quite entirely self-contained—it can leak ROS, leading to a cascading effect of neuronal cell death in the immediately neighboring tissue. Though this knock-on effect of neuroapoptosis is likely quite spatially limited, the dissemination of ROS following necrotic cell death is far more extensive. ROS-induced necrosis of neurons, such as following the accumulation of hydrogen peroxide [136] and/or peroxynitrite [137] and the alteration of glutamate signaling [138], leads to a rapid and unregulated death sequence that can release the contents of the cell. We would like to place particular emphasis on peroxynitrite in this process as it is very reactive, while also being characterized by a relatively long biological half-life. Therefore, peroxynitrite generation would undoubtedly cause severe neuronal damage, which would, in turn, yield inflammation, which is known to also upregulate ROS production [55]. Since ROS are capable of both diffusing across and destroying membranes, it is intuitive that apoptotic, and particularly necrotic, neuronal cell death can cascade and likely accounts for a substantial amount of anesthesia-induced cell death.

Oxidative stress during anesthesia seems to depend on the type of chosen anesthetics. Each anesthetic has different pharmacokinetic and pharmacodynamic properties that can define the level of oxidative stress. For example, propofol is very common in the pediatric population and requires relatively higher doses because of the higher clearance rate of the drug. Multiple reports consider propofol to have an antioxidative effect due to an increase in antioxidant production following its administration [139,140,141,142,143,144,145]. However, this effect may be a response to a propofol-induced increase in ROS production. Evidence for this was reported by a study that measured an increase in total oxidant status of blood plasma after propofol administration [146]. In addition, a separate study on propofol observed higher malondialdehyde concentrations, an indicator of lipid oxidation damage, even compared with subjects who underwent desflurane anesthesia [147] (which is also known to increase the production of ROS [148]). However, the mechanisms of propofol’s influence on the production of ROS and redox balance remain to be fully established.

Similar interactions with redox balance have also been observed with ketamine. Some reports, related to animal models of depression, have described ketamine to have antioxidative properties. Ketamine, used as an antidepressive treatment, increased the post-treatment production of superoxide dismutase and catalase in [149] and reduced the production of ROS in [150]. However, the latter study utilized a low dose (3 mg/kg intraperitoneally) of ketamine. In contrast, higher doses in animal studies (e.g., 30–75 mg/kg intraperitoneally or 200 mg/kg, over a 3 h intravenous infusion) have shown to increase the production of ROS and reduce superoxide dismutase and catalase activity [151,152,153,154,155]. Studies that have investigated the mechanisms of ketamine-induced toxicity exhibit a common theme—ketamine antagonism of calcium-permeable receptors dysregulates intracellular calcium levels, subsequently leading to mitochondrial dysfunction (complex I) and the overproduction of ROS (most notably, H_2_O_2_) [156,157]. Moreover, the oxidative stress that is affiliated with ketamine can induce autophagy and apoptosis. This, in turn, outlines a serial pathway by which the use of ketamine, for sedative or anesthetic purposes, contributes to the neuronal cell loss.

It is also possible that volatile anesthesia-induced hypoglycemia plays a role in the production of oxidative stress [158,159,160,161]. The low availability of intracellular glucose during hypoglycemia is thought to produce oxidative stress by eliciting increased ROS production via a combination of inflammation [162], endothelial cell dysfunction [163], and dysregulated mitochondrial function, characterized by the downregulation of both catalase and superoxide dismutase, with the simultaneous upregulation of lipid peroxidation [164]. However, while hypoglycemia may contribute to oxidative stress during anesthesia, the substantial drop in glucose demand due to the global reduction of neuronal activity [116,165] must also be considered, as this would lessen the severity of hypoglycemic consequences within neurons. For this reason, it seems to be more convincing that the neonatal anesthesia-related oxidative stress occurs due to other factors, such as hyperoxia, altered neuronal activity, and neuroapoptosis.

Overall, depending on the anesthetic agent and circumstances of anesthesia administration, numerous ROS are produced, leading to oxidative stress. Unfortunately, many of the precise molecular interactions regarding how anesthetic agents engender elevated levels of ROS in the neonatal brain remain to be elucidated. However, some human studies have reported markers of lipid peroxidation and DNA damage from oxidative stress to be significantly higher after general anesthesia compared with baseline levels taken before its administration [166,167,168,169]. A leading hypothesis of the molecular interactions regarding how this can manifest was proposed by Ni et al. [170]. In the study, the investigators reported that isoflurane induced oxidative stress in H4 human neuroglioma cells, leading to caspase-3 activation, causing DNA damage, with simultaneous DNA repair via lowered p53 levels. This is a novel perspective and is likely another contributing factor to the levels of oxidative stress in neonatal anesthesia that manifest in conjunction with altered signaling, hyperoxia, and neuronal cell death.

An important limitation of human studies are the additional sources of systemic oxidative stress that result from invasive procedures. Human studies of anesthesia often are not conducted in isolation without an external need for anesthesia administration. Damage to tissue, inflammation, and ischemic reperfusion injury are common consequences of surgical interventions and are each known to promote oxidative stress through the increased production of ROS [15,55]. It seems that the predominant pathway of soft tissue damage and oxidative stress appears to be from the cyclical behavior of the inflammatory response, where disturbed microvasculature leads to the oxygen supply being insufficient for oxygen demand, which is exacerbated by the elevated presence of infiltrating immune cells, thereby causing mitochondrial dysfunction and the overproduction of ROS, which further promotes the inflammatory response [55].

The direct relationship between oxidative stress and soft tissue damage is supported by the observation of increased oxidative stress markers in open cholecystectomy surgery compared with minimally invasive laparoscopic surgery [171]. In the case of cardiac interventions, ROS have been implicated in complications, such as arrhythmias, transient mechanical dysfunction, and cell death [172]. Moreover, some procedures involve the use of tourniquets to reduce blood flow to the operating site and have shown to be associated with longer healing periods [173]. It is possible that ROS may accumulate in the hypoxic tissue, which are then released with reperfusion, contributing to systemic oxidative stress. However, additional studies are needed to quantify the role of oxidative stress in healing and neuronal damage in such circumstances.

Since neonatal anesthesia in humans is not performed without procedural necessity, animal models have been instrumental in demonstrating anesthesia-related oxidative stress, as the previously discussed animal models predominantly administered neonatal anesthesia independent of invasive surgery. To recapitulate, these studies have consistently shown signs of anesthesia-induced oxidative stress in neonates. Therefore, depending on the surgical procedure, the oxidative stress that ensues is likely the result of the combined consequences of anesthesia administration and the trauma of the surgery itself. Unfortunately, to date, the relative contributions of the specific surgical procedure and anesthesia to the overall level of oxidative stress are yet to be systematically modeled.

### 3.3. Oxidative Stress Following Anesthesia

Apoptosis and oxidative stress, which are directly related to general anesthesia, end within a few hours/days [165] after the end of anesthesia administration. These events represent an initial phase of anesthesia-induced neurotoxicity. We would like to clarify that the role of ROS and oxidative stress in anesthesia-induced neuroapoptosis does not likely extend to the post-anesthesia period beyond a few days. However, after that, the developmental changes, associated with initial anesthesia events, commence. They include restructuring of the neuronal network to compensate for the loss of neurons and changes in cellular morphology, which manifest, for example, as reduced dendritic branching and shorter dendritic lengths in the hippocampus [124]. Although such processes can generally affect brain functions, including learning and memory, we would like to concentrate on specific neuronal changes that can potentially produce oxidative stress, as outlined in Figure 1. These changes involve the development of the neurovascular unit, which can also become compromised [124,174].

Normal neurovascular interactions are critical for healthy brain functions. The canonical perspective of neurovascular interactions revolves around focal increases in cerebral blood flow in response to transient increases in neuronal activity. Although the relationship between neuronal activity and vascular responses has been well established and is the basis of widely used techniques, such as functional magnetic resonance imaging (fMRI), a complete understanding of how neuronal activity elicits such a response remains to be achieved.

A broad variety of pathways have been shown to be involved in this process. Since the increased blood flow results from an increase in neuronal activity, the role of the glutamatergic and astrocytic pathways in neurovascular coupling has been the focus of much research. Specifically, multiple studies have proposed specific byproducts of these pathways (e.g., nitric oxide, arachidonic acid metabolites, and both calcium and potassium ions) participating in neurovascular interactions [175,176,177,178].

Neuronal excitatory responses are accompanied by inhibitory activity, which both spatially and temporally confines areas of excitation. This process highlights the potential role of GABA (the brain’s most prevalent inhibitory neurotransmitter) and GABAergic interneurons in neurovascular interactions. Indeed, GABA receptors have shown to be located along arterioles [179] where GABAergic interneurons make direct functional connections and are capable of dilating arterioles [180,181]. Through these means, GABAergic signaling ensures two main effects: (1) increasing local oxygen delivery and (2) decreasing neuronal consumption during various types of stimulation [182]. Thus, numerous cell types are required for the normal maintenance of oxygen levels in the brain, and if any were to be disrupted, it could engender dysfunctional neurovascular interactions.

The pathology of neurons involved in neurovascular interactions can lead to neurovascular deficiency. Without sufficient signaling to the microvasculature, the surrounding brain tissue can experience localized areas of hypoxia, leading to an increase in ROS concentrations, such as nitric oxide and peroxynitrite. We would like to differentiate this type of hypoxic injury from ischemic strokes, where a few or a single area of damage with extensive necrosis and apoptosis in the associated region appears [183,184]. However, areas of localized hypoxia due to neurovascular deficiency still were shown to have significantly elevated levels of neuronal apoptosis in [185,186].

In the postnatal period, both glutamatergic and GABAergic systems undergo critical stages of development. For example, in vitro studies have shown that GABA may first be excitatory in nature, before developing the mature function of inhibition [187,188,189,190], and animal models have found that GABAergic synapses become functionally active before glutamatergic ones [191,192]. This phenomenon is needed to activate several signaling mechanisms that influence dendritic development, cell migration, proliferation, and synaptogenesis [193,194]. Similarly, the glutamatergic system is also an integral part of synaptogenesis, migration, and plasticity in the neonatal brain [195]. During development, the different subunits of glutamate receptors are expressed at varying rates [196]. Thus, the widespread neuroapoptosis and cellular damage resulting from neonatal anesthesia during this period can negatively affect the development of these systems, such that their full mature functioning, including participation in neurovascular interactions, is not achieved.

Neonatal anesthesia can detrimentally affect the normal development of neurons [10]. This consequence of neonatal anesthesia has been proposed to become observable much later when damaged neuronal networks cannot obtain normal maturity [10]. If these networks are responsible for neurovascular coupling, neuronal oxygen demand will exceed oxygen supply and focal and disseminated areas of hypoxia may manifest [10]. Brain hypoxia has been shown to induce oxidative stress by simultaneously increasing ROS production [197,198,199,200,201] and weakening the antioxidative system [202,203]. The lower levels of oxygen leads to a reduction in mitochondrial ATP production due to the interference and inhibition of complexes I and III in the electron transport chain [204]. Such a change has subsequently been shown to alter the function of complex II, causing it to overproduce ROS [205].

The type of oxidative stress discussed in this section can appear following a significant delay (years in humans) and last a considerably long time compared with the immediate oxidative stress during neonatal anesthesia.

## 4. Conclusions

Oxidative stress is abundantly observed during neonatal anesthesia across multiple anesthetic agents and methods of administration. This is due to the sensitive and relatively weak antioxidant defense system in the neonatal brain. The consequences of volatile anesthetics have been reported to be quite similar and consistent in producing oxidative stress. Other anesthetics (e.g., propofol and ketamine) tend to have less clear influences, such that low doses may stimulate the antioxidant system, while higher anesthetic doses can induce oxidative stress.

The types of oxidative stress caused by neonatal anesthesia can be grouped into three distinct categories (Figure 1). First, there is the immediate oxidative stress, which results from anesthesia and procedural stressors on redox balance. This type of oxidative stress exists throughout the administration of anesthesia and shortly after its cessation (likely lasting no more than a couple hours). As previously discussed, numerous studies have observed significantly elevated levels of ROS and reduced concentrations of antioxidants in the brain during anesthesia—likely due to the combination of hyperoxia from supplemental oxygen, anesthesia-induced apoptosis, and burst suppression that affects neurovascular interactions. Alternative sources of oxidative stress during anesthesia have also been proposed, such as hypoglycemia; however, additional research is recommended for such sources to be more strongly considered. As regards the short-term effects (which refer to the days, weeks, or even months after anesthesia in children), there does not appear to be any strong pathophysiological basis for oxidative stress to occur during this time.

However, there appears to be long-term oxidative stress. Since anesthesia causes selective neuronal apoptosis, neurovascular coupling may not develop to sufficiently support mature oxygen demand. There is a critical period of development for both excitatory and inhibitory signaling, which can become compromised by general anesthesia. Indeed, the initial apoptosis can involve cells that are supposed to participate later in neurovascular coupling. Even cells that survive this initial wave of neonatal anesthesia-induced apoptosis can exhibit damage. The consequences of such insults likely take many years to develop and can lead to focal and disseminated areas of oxidative stress due to relative hypoxia in local networks from a lack of sufficient oxygen supply during increased neuronal activity. Since the GABAergic system participates in both restricting metabolic demand of oxygen and promoting increases in its delivery, the targeted effect of anesthetics on interneurons likely exacerbates the long-term consequences. These findings support the conclusions of large observational studies that report learning and behavioral deficiencies after neonatal anesthesia becoming particularly visible in later childhood. However, it should be noted that a clear picture of all potential deficits and the likelihood of such deficits occurring are yet to be fully established. For example, a recent paper reported that children exposed to anesthesia within the first 36 months of life reported lower IQ scores compared with the control group, yet the mean score of the anesthesia group was still above the expected population average [206]. The authors here note that other factors, such as full-term birth, may have influenced these results. Thus there is likely a vast range of factors that modulate the development of this type of oxidative stress, including the duration of initial anesthesia, the invasiveness of the concurrent surgical procedure, the location of damaged cells, their relative number, and the relationship between environmental stimulation and affected local networks, and so forth.

The time period of neonatal anesthesia outlined in this review is not an all-encompassing period of high susceptibility to anesthesia toxicity. Maternal anesthesia also presents its own significant risks. Graf et al. [207] noted that the effects of oxidative stress from supplemental oxygen and maternal anesthesia in premature infant mice pups led to the observation of significantly lower oligodendrocyte numbers. These findings are supported by a study on the fetal nonhuman primate brain, which reported widespread oligodendrocyte and neuronal apoptosis after maternal anesthesia [208]. Since anesthesia was administered to the mother, it is not fully understood to what extent anesthesia and supplemental oxygen each contribute to the harmful effects on neuronal development, though these findings suggest supplemental oxygen to exacerbate the negative consequences.

Reducing perioperative oxidative stress not only has shown to improve surgical recovery rates [209] but may also mitigate some of the consequences on the neonatal brain. In order to assess the direct effects of oxidative stress on the brain, it is likely necessary for advancements to be made in perioperative monitoring of oxidative stress. Current standards of measuring oxidative stress often involve testing ex vivo blood or urine samples to directly assess global concentrations of ROS or by the indirect means of measuring the biochemical markers of elevated ROS. Typical techniques involve chemiluminescence assays, flow cytometry, total oxidant or antioxidant assays [210], or the human heme oxygenase-1 (HO-1) assay [211]. The in vivo techniques have shown to be highly efficacious in testing for the presence of global oxidative stress; however, the oxidative stress produced by neonatal anesthesia, particularly the long-term pathway presented above, may be highly tissue specific and spatially limited, which would not yield notable changes in global levels of ROS. In response to the difficulty of measuring local oxidative stress, various MR modalities have been developed. For example, MR spectroscopy, which has been shown to be able to evaluate the concentration of specific biomolecules after neonatal anesthesia in the hippocampus [124], may serve as an indirect approach to measure oxidative stress (for example, by evaluating the level of the antioxidant, glutathione). Additionally, the recently developed technique of QUEnch-assiSTed or QUEST MRI has demonstrated its ability to measure the excessive production of ROS in various tissues [211,212,213,214], including the brain [215]. However, given the nature of these techniques, spatial resolution can be considered a hurdle that must first be overcome for widespread clinical application to be possible. Therefore, in the meantime, we anticipate animal models that use invasive techniques to constitute a significant proportion of the methodologies in the near future to further investigate the relationship between neonatal anesthesia and oxidative stress.
